# Efficient base- and ligand-free palladium catalysed *O*-arylation of phenols in choline chloride:triethanolamine as a reusable deep eutectic solvent

**DOI:** 10.1098/rsos.240045

**Published:** 2024-06-05

**Authors:** Fatemeh Abbasi, Ali Reza Sardarian

**Affiliations:** ^1^ Chemistry Department, College of Sciences, Shiraz University, Shiraz 71946-84795, Iran

**Keywords:** deep eutectic solvents, carbon–oxygen bond formation, choline chloride, triethanolamine, reusable solvent/catalyst system

## Abstract

In this paper, we present a novel and sustainable approach using choline chloride:triethanolamine as a green, efficient and reusable deep eutectic solvent (DES) for Pd-catalysed *O*-arylation reactions with Pd/BaSO_4_ (10%). By using the unique properties of DESs, we successfully achieved C–O bond formation without the need for additional solvents, bases and ligands. This solvent/catalyst system ([ChCl][TEA]_2_) functioned as a dual catalyst and solvent system, enabling fast and environmentally friendly C–O bond formation from phenol derivatives and electron-deficient aryl halides, leading to remarkable yields under mild reaction conditions. To identify and characterize this DES, we employed differential scanning calorimetry, thermogravimetric analysis, Fourier-transform infrared spectroscopy, refractive index, viscosity, the potential of hydrogen (pH) and conductivity measurements. One of the remarkable advantages of this DES system is its exceptional stability. This solvent/catalyst system exhibited high stability throughout the reaction cycles, showing no significant loss of activity. As a result, this DES and catalyst (Pd/BaSO_4_ (10%)) can be easily recycled and re-used for up to three consecutive cycles, making it an economically and environmentally attractive option for organic reactions. Our approach offers several key benefits, including simple catalyst preparation, quick reaction times and excellent production efficiency.

## 1. Introduction

The principles of green chemistry emphasize the importance of stable, affordable and reusable catalysts, and also using non-toxic solvents to reduce expenses and waste in chemical processes [1,2]. As a result, there have been numerous recent efforts to introduce these catalysts. Ionic liquids (ILs), for instance, are frequently used in organic reactions because of their notable characteristics such as low vapour pressure, non-corrosiveness, non-flammability and relatively good thermal and chemical stability [3]. Also, deep eutectic solvents (DESs), which represent a class of ILs, exhibit dual functionality as solvents and catalysts [4]. They function as green and environmentally friendly solvents in numerous organic reactions, including coupling reactions [5], hydrogenation, Diels–Alder reaction, oxidation, etc. [6,7].

Due to their diverse applications in the synthesis of medicines, polymers and natural products, carbon-heteroatom (O, N and S) bonds are regarded by synthetic chemists as a popular and effective approach [8,9]. Transition metal-catalysed methodologies for the formation of carbon-heteroatom bonds, particularly C–O bonds, have been actively pursued in synthetic organic chemistry over the past few decades [10].

**Scheme 1.**
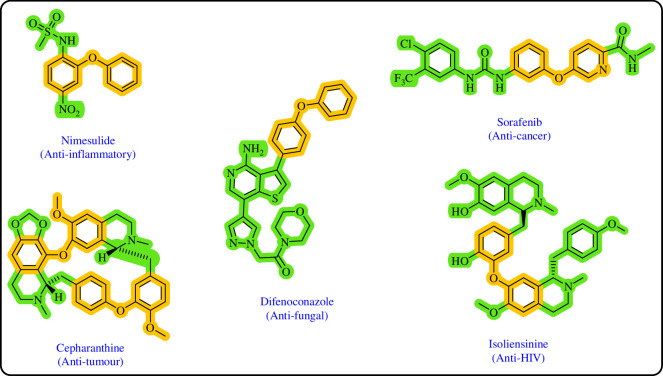
Some structures of commercial drugs with the diaryl ether motif.

Diaryl ethers, an important class of organic compounds, are present in many biologically active natural products and play a critical role in the chemical industry and medicinal chemistry. Various diaryl ethers have demonstrated specific and significant biological and medicinal activities, such as anti-inflammatory, anti-viral, anti-bacterial and anti-cancer properties, along with applications as commercial dyes (scheme 1) [11].

The natural abundance and biological potential of numerous medicinal derivatives containing ether functionalities have earned significant attention from researchers in recent years [9]. In 1901, for the first time, diaryl ethers was synthesized via the Ullmann C–O coupling reaction of phenols with aryl halides in the presence of copper powder [12]. As this process demands difficult reaction conditions, such as lengthy reaction durations, high temperatures (greater than 200°C) and stoichiometric quantities of copper, it has a limited range of applications [13,14]. Substantial research efforts have focused on overcoming these limitations, resulting in significant advancements in the field. The development of catalytic versions of the original copper salt-mediated C–O coupling procedures, as well as the exploration of alternative metals such as palladium [15], iron [16], cobalt [17] or nickel [18,19] have proven to be particularly intriguing and innovative. The advent of Pd(0) and Cu(I)-based catalytic systems has led to breakthroughs in coupling reactions [20–22]. Although Pd/BaSO_4_ has been employed as a catalyst in the Stille [23] and Heck reactions [22], C–O coupling has never taken place using this catalytic system. Because the supported Pd nanoparticles have varying basicity (acidity), diameters and dispersions [24], among other properties, it is expected that this source may exhibit various behaviours.

**Scheme 2.**
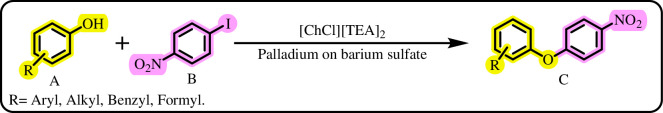
Pd-catalysed coupling between 1-iodo-4-nitrobenzene and phenols in [ChCl][TEA]_2_ as a DES.

In this study, we present for the first time a base- and ligand-free protocol using Pd/BaSO_4_ as the source of Pd(0) in the C–O coupling reaction between aryl halides and phenols using a DES as the solvent/catalyst system, without needing additional additives (scheme 2).

## 2. Experimental section

### 2.1. General

All chemicals used in this study were commercially available and employed without further purification. On a Bruker-400 MHz nuclear magnetic resonance (NMR) spectrometer, ^1^H and ^13^C NMR spectra were recorded at 400 (^1^H NMR) and 100 (^13^C NMR) MHz. The signals of the NMR spectra are reported at a downfield of TMS (trimethylsilyl chloride) (δ 0.00) as an internal standard in parts per million (ppm). Coupling constants (J) were expressed in Hertz (Hz). Fourier-transform infrared (FT-IR) spectra of the materials were obtained at room temperature using potassium bromide pellets and a Shimadzu FT-IR-8300 device, in the 400–4000 cm^−1^ range. Thermo Finnegan Flash EA-1112 CHNS rapid elemental analyser was used to perform the analyses of the C, H, N and S elements. An Electrothermal 9100 melting point device was used to determine melting points. The characterization of all products was accomplished by employing FT-IR, ^1^H and ^13^C NMR spectroscopy, elemental analysis (CHNS) and comparison with the literature data. A TA Instruments TGA Q600 instrument was used to perform thermogravimetric analysis (TGA)/differential thermal analysis (DTA) for DES to examine the DES’s thermal stability. Differential scanning calorimetry (DSC) measurements of the [ChCl][TEA]_2_ mixture were conducted using a TA Instruments DSC Q600 instrument to analyse the phase transitions. Metrohm devices (660 for conductivity and 780 for pH) were used to determine the conductivity and pH of the DES. An Anton Paar MCR-302 rheometer was used to measure the solvent/catalyst system’s viscosity, and an Abbe-RefraKtometer AR4 was used to calculate the refractive index.

### 2.2. The synthesis of [ChCl][TEA]_2_ deep eutectic solvent [25]

A mixture of ChCl (1 mmol, 0.139 g) and triethanolamine (TEA) (2 mmol, 0.264 ml) was blended and heated in an oil bath at 90°C until a homogeneous colourless liquid emerged. The mixture was employed after cooling at room temperature without additional purification.

### 2.3. Procedure for *O*-arylation of phenols (C1–17)

A total of 1.2 mmol of aryl halide, 1 mmol of phenol derivative and 0.006 g of Pd /BaSO_4_ (10%) were poured into a round-bottom flask containing 5 mmol (3 ml) of the DES ([ChCl][TEA]_2_). Under vigorous stirring, the aforementioned mixture was heated at 90°C for 3 h. Thin layer chromatography was used to follow the reaction’s progress (ethyl acetate/*n*-hexane, 1:5). Once the reaction was complete, the mixture was allowed to cool at room temperature. Then, 5 ml of water and 5 ml of ethyl acetate were added to the reaction mixture and blended strongly for 5 min at room temperature. Next, the desired mixture was transferred to the decanter funnel, and after slowly mixing the contents inside the funnel, DES, palladium on barium sulfate and organic phase were placed in the decanter funnel as separate phases that were easily separated from each other. Finally, the aqueous solution containing DES was extracted with ethyl acetate (3 × 5 ml) to separate the remaining organic substances in this phase. The mixed organic phase was filtered after being dried with anhydrous MgSO_4_ as a drying agent. After filtration, the organic layer was concentrated using a rotary evaporator to produce the corresponding crude. In the end, recrystallization with ethanol/*n*-hexane produced the pure products. By using FT-IR, CHNS analyses as well as ^1^H and ^13^C NMR, these products' identification and purity were verified. The recovered [ChCl][TEA]_2_ was heated under a reduced vacuum for 40 min at 70°C and then used again for the next cycles. It should be noted that Pd/BaSO_4_ was readily isolated from the reaction mixture and that, following isolation, it was employed three times in a row without noticeably losing any of its activity.

## 3. Results and discussion

### 3.1. Deep eutectic solvent characterization

#### 3.1.1. Fourier-transform infrared spectroscopy analysis

**Figure 1.**
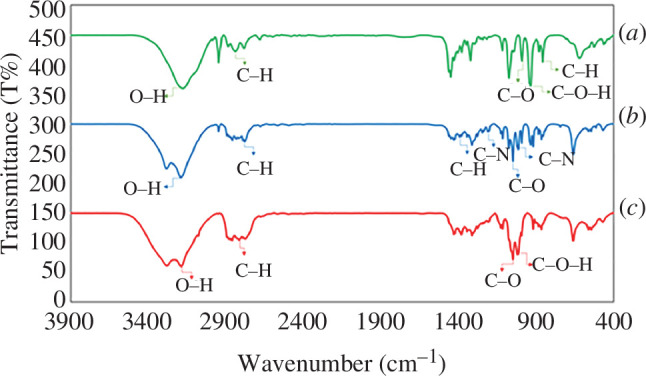
FT-IR spectra of (*a*) pure ChCl, (*b*) pure TEA and (*c*) the DES ([ChCl][TEA]_2_).

FT-IR analysis was used to verify the existence of bonds or functional groups of atoms in compound molecules as well as to investigate how molecules interact. The FT-IR spectra of the final DES system and its components, ChCl and TEA, were recorded using Shimadzu’s FT-IR-8300 spectrophotometer in the range of 400–4000 cm^−1^ (figure 1). The first spectrum (figure 1*a*) represents the FT-IR spectrum of pure ChCl, the second spectrum (figure 1*b*) corresponds to pure TEA and the third spectrum (figure 1*c*) illustrates the FT-IR spectrum of the DES. Hydroxyl groups appear as strong bonds above 3000 cm^−1^ in the FT-IR spectrum. In the ChCl spectrum (figure 1*a*), the bending and stretching vibrations of C–H bonds are observed at 873 and 2936 cm^−1^, respectively. Additionally, the bands at 1134 and 1088 cm^−1^ can be attributed to C–O and C–O–H stretching vibrations, respectively. As TEA is a tertiary amine, it lacks the N–H stretch, and the corresponding C–N stretch is observed at 1224 cm^−1^. The characteristic peaks at 1032, 1066, 1408 and 2866 cm^−1^ correspond to the *ν* (C–N), *ν* (C–O), (C–H) and δ (C–H) groups of the TEA molecule [26]. Figure 1*c* demonstrates that [ChCl][TEA]_2_ exhibits absorption bands corresponding to ChCl and TEA.

#### 3.1.2. Thermogravimetry analysis and differential thermal analysis

**Figure 2.**
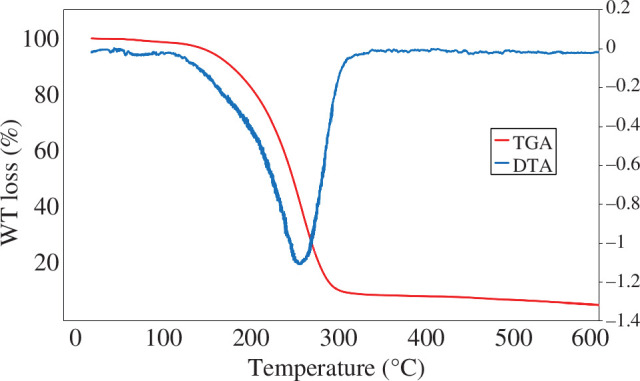
TGA and DTA of [ChCl][TEA]_2_ system with a heating rate of 20°C min^–1^.

We investigated the thermal behaviour of the DES system using the TGA and DTA plots in the temperature range of 0–600°C in a nitrogen atmosphere with a heating rate of 20°C min^−1^ (figure 2). The initial weight loss observed at around 100°C is attributed to the evaporation of adsorbed water molecules and is common in many DESs [27]. The maximum weight loss has happened at the range of 125–341°C as a result of complete decomposition of the DES.

#### 3.1.3. Differential scanning calorimetry analysis

**Figure 3.**
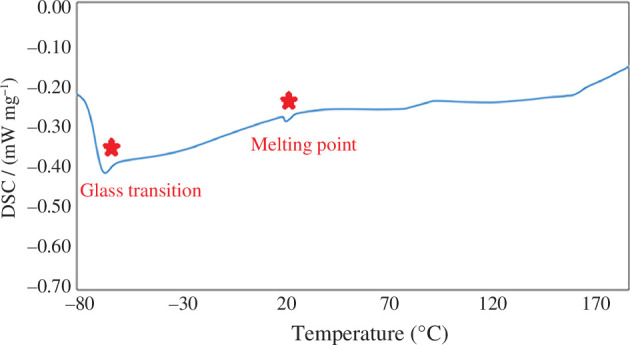
DSC analysis of [ChCl][TEA]_2_ with a heating rate of 15°C min^–1^.

A crucial feature of DESs is their thermal property. DSC analysis was studied for DES behaviour and phase transition study of [ChCl][TEA]_2_. This analysis was used to determine the glass transition temperature (Tg) and melting point using a differential thermal analyser from −80 to 180°C with a heating rate of 15°C min^−1^ after cooling samples to −80°C in an aluminium pan. The DSC curve in figure 3 reveals a glass transition occurring at approximately −66.55°C and a melting point of the crystalline phase at around 18.92°C. This drop in the final melting point is the evidence of the desired DES’s deep eutectic behaviour (figure 3).

#### 3.1.4. Cyclic voltammetry analysis

**Figure 4.**
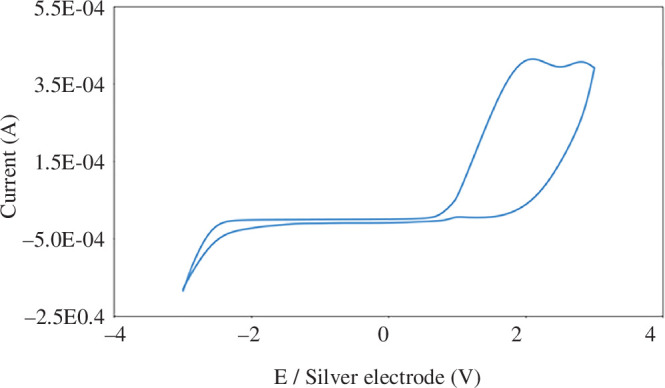
Cyclic voltammogram of the ChCl:TEA system at a scan rate of 50 mV s^−1^. Working electrode (Pt), reference electrode (silver wire) and counter electrode (Pt).

Using a cyclic voltammogram, the oxidation–reduction potential range, reversibility or irreversibility and electrochemical behaviour of the corresponding DES ([ChCl][TEA]_2_) were all determined. The working electrode was polished with alumina (Buehler, Lake Bluff, IL, USA), placed on a clean polishing cloth, washed twice with distilled water and ethanol and dried with lint-free paper towels. In this test, a platinum rod electrode works as the counter electrode, a glassy carbon electrode operates as the working electrode and a silver wire acts as the reference electrode in the absence of a supporting electrolyte at room temperature with a scanning speed of 50 mV s^−1^. Notably, larger and sharper oxidation peaks were observed at 2.16 and 2.86 V during the experiment (figure 4).

#### 3.1.5. Viscosity analysis

**Figure 5.**
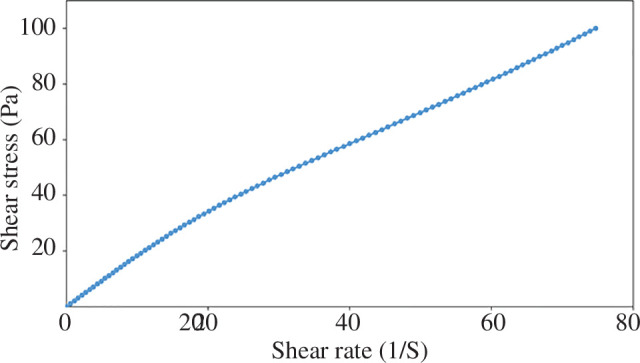
Shear stress in terms of shear rate for [ChCl][TEA]_2_ at 25°C.

DESs are known to exhibit significantly higher viscosity compared with conventional molecular solvents due to the presence of hydrogen bonding. The viscosity of the synthesized DES was measured at 25°C, yielding a value of 1881 mPa s^−1^. To determine the fluid behaviour, the shear stress of the DES was analysed in terms of its shear rate. The results indicated that the desired DES exhibits Newtonian behaviour (figure 5).

#### 3.1.6. Ionic conductivity analysis

The conductivity and fluidity of ILs are strongly correlated. The ionic conductivity of a DES is influenced by factors such as the degree of ion separation, the number of charges and ion mobility [28]. Polar ionic liquids (PILs) often exhibit limited ion mobility due to their relatively high viscosity, leading to lower conductivity. The addition of water to ILs enhances their conductivity by reducing viscosity [29]. The ionic conductivity of the synthesized DES was measured using a Solartron 1260 instant response analyser in a cell with a platinum electrode over a temperature range of 25–60°C (Farnborough, UK). The relationship between the ionic conductivity of the DES and temperature is presented in table 1. As expected, the electrical conductivity of the corresponding DES increased with rising temperature.

**Table 1.** Evaluation of ionic conductivity in different temperatures for [ChCl][TEA]_2_.

temperature (˚C)	conductivity (ms cm^−1^)
25	2.2
30	2.3
35	2.5
40	2.8
45	3.0
50	3.2
55	3.4
60	4.0

#### 3.1.7. Density, refractive index and potential of hydrogen analysis

In general, DESs are typically highly dense. Various factors can influence DES density. The hole theory can explain the higher densities observed in DES systems, considering the presence of voids similar to other ionic materials. When different hydrogen bond donors (HBDs) and hydrogen bond acceptors (HBAs) are combined, the average pore radius may decrease, resulting in increased DES density. The type of material also plays a role. For instance, when carboxylic acids are used as HBDs in type III of DESs, the number of carbons in the chain is typically inversely correlated with density. Longer chain lengths lead to higher molar mass and volume, causing a decrease in density due to increased molar volume.

In this study, the high density of the [ChCl][TEA]_2_ system (1.55 g cm^−3^, 25°C) can be attributed to the strong intermolecular hydrogen bonding network between TEA and ChCl, which provides TEA with strong cohesive energy. The refractive index of [ChCl][TEA]_2_ was measured to be 1.29 Pa s^−1^. The pH of the DES (10^−2^ M, [ChCl][TEA]_2_) was determined to be 9.1 using a pH meter (model 780) at room temperature, indicating the alkaline nature of the corresponding DES.

#### 3.1.8. Optimization of the reaction conditions

After the completion of identification testing for the DES and ensuring the formation of DES, this catalyst was used for coupling of 1-iodo-4-nitrobenzene with phenolic derivatives. To determine the optimal conditions for this reaction, various DESs derived from ChCl or *N*-benzyl-2-hydroxy-*N*,*N*-dimethylethan-1-aminium chloride (NBHDMACl) [30] as the HBA and different HBDs such as urea, thiourea, glycerol, TEA, benzoic acid (BA) and metal halides (MCl) (M = Fe, Zn, Sn, Ni and Cu) were evaluated (table 2, entries 1–20). The results indicate that the best yield was achieved with the [ChCl][TEA]_2_ DES (table 2, entry 4). Lower molar ratios of ChCl:TEA (1:1, 1:1.5) led to reduced effectiveness, probably due to the DES’s higher viscosities (table 2, entries 21 and 22). Higher molar ratios of ChCl:TEA (1:3 and 1:4) did not improve the yields of the aryl ether (table 2, entries 23 and 24). The role of the DES was further demonstrated by conducting the reaction without [ChCl][TEA]_2_, which resulted in no formation of the desired product even after 24 h (table 2, entry 25). Additionally, when TEA was used alone, the desired product was obtained with a relatively low yield (table 2, entry 26). Also, when choline chloride is present alone, the desired reaction does not occur at all (table 2, entry 27). Subsequently, different amounts of the [ChCl][TEA]_2_ solvent/catalyst system were tested (table 2, entries 28–31). The effect of palladium as a catalyst was also investigated, revealing that no product was formed in its absence (table 2, entries 32–34), and the best efficiency was achieved with 0.006 g of the catalyst (table 2, entry 4). Moreover, it should be noted that the effects of different molar ratios of *m*-cresol and 1-iodo-4-nitrobenzene in the model reaction were investigated. It was observed that in different molar ratios (table 2, entries 35 and 36), large amounts of raw materials remained intact and made no significant impact on the amount of desired product. The best result was obtained when the molar ratio of 1:1.2 for *m*-cresol:1-iodo-4-nitrobenzene was used in this reaction (table 2, entry 4). Furthermore, it was observed that the coupling reaction efficiency was sensitive to the reaction temperature and the product yield decreased significantly with decreasing temperature, reaching to its highest yield at a reaction temperature of 90°C (table 2, entries 37–40). Next, the reaction time was investigated (table 2, entries 41–44), and it was determined that the highest yield of diaryl ether (at 90°C in 3 h) was achieved by reacting 1-iodo-4-nitrobenzene (1.2 mmol) and *m*-cresol (1 mmol) in the presence of 5 mmol (3 ml) of [ChCl][TEA]_2_ (table 2, entry 4).

In summary, the optimization of the reaction conditions demonstrated the crucial role of the [ChCl][TEA]_2_ DES, catalyst, molar ratios, temperature and reaction time in achieving the desired product yield.

**Table 2.** Optimization of reaction parameters for one-pot synthesis of diaryl ethers.
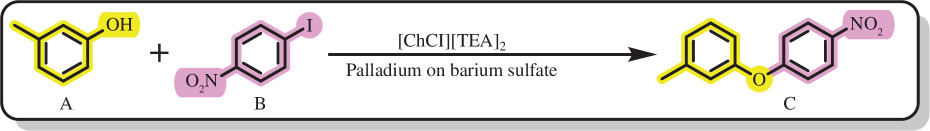

entry	molar ratio A:B	Pd/BaSO_4_ (10%) (g)	DES	DES (mmol)	molar mass of DES (g mol^−1^)^a^	T (^o^C)	time (h)	yield (%)^b^
1	1:1.2	0.006	[ChCl][Urea]_2_	5	86.58	90	3.0	78
2	1:1.2	0.006	[ChCl][Thiourea]_2_	5	97.29	90	3.0	81
3	1:1.2	0.006	[ChCl][Glycerol]_2_	5	107.93	90	3.0	84
**4**	**1:1.2**	**0.006**	**[ChCl][TEA]_2_**	**5**	**146**	**90**	**3.0**	**93**
5	1:1.2	0.006	[ChCl][BA]_2_	5	127.95	90	3.0	43
6	1:1.2	0.006	[ChCl][FeCl_3_]_2_	5	132.94	90	3.0	47
7	1:1.2	0.006	[ChCl][ZnCl_2_]_2_	5	136.17	90	3.0	49
8	1:1.2	0.006	[ChCl][SnCl_2_]_2_	5	154.67	90	3.0	46
9	1:1.2	0.006	[ChCl][NiCl_2_]_2_	5	172.94	90	3.0	53
10	1:1.2	0.006	[ChCl][CuCl_2_]_2_	5	137.41	90	3.0	56
11	1:1.2	0.006	[NBHDMACl][Urea]_2_	5	111.95	90	3.0	64
12	1:1.2	0.006	[NBHDMACl][Thiourea]_2_	5	122.65	90	3.0	67
13	1:1.2	0.006	[NBHDMACl][Glycerol]_2_	5	133.30	90	3.0	71
14	1:1.2	0.006	[NBHDMACl][TEA]_2_	5	171.37	90	3.0	75
15	1:1.2	0.006	[NBHDMACl][BA]_2_	5	153.32	90	3.0	41
16	1:1.2	0.006	[NBHDMACl][FeCl_3_]_2_	5	180.04	90	3.0	45
17	1:1.2	0.006	[NBHDMACl][ZnCl_2_]_2_	5	162.71	90	3.0	47
18	1:1.2	0.006	[NBHDMACl][SnCl_2_]_2_	5	198.30	90	3.0	44
19	1:1.2	0.006	[NBHDMACl][NiCl_2_]_2_	5	158.31	90	3.0	51
20	1:1.2	0.006	[NBHDMACl][CuCl_2_]_2_	5	161.54	90	3.0	54
21	1:1.2	0.006	[ChCl][TEA]	5	144.40	90	3.0	87
22	1:1.2	0.006	[ChCl][TEA]_1.5_	5	145.36	90	3.0	89
23	1:1.2	0.006	[ChCl][TEA]_3_	5	146.79	90	3.0	91
24	1:1.2	0.006	[ChCl][TEA]_4_	5	147.28	90	3.0	91
25	1:1.2	0.006	none	5	0	90	3.0	0
26	1:1.2	0.006	TEA	—	—	90	3.0	61
27	1:1.2	0.006	ChCl	—	—	90	3.0	0
28	1:1.2	0.006	[ChCl][TEA]_2_	3	146	90	3.0	83
29	1:1.2	0.006	[ChCl][TEA]_2_	4	146	90	3.0	86
30	1:1.2	0.006	[ChCl][TEA]_2_	6	146	90	3.0	89
31	1:1.2	0.006	[ChCl][TEA]_2_	7	146	90	3.0	86
32	1:1.2	none	[ChCl][TEA]_2_	5	146	90	3.0	0
33	1:1.2	0.004	[ChCl][TEA]_2_	5	146	90	3.0	79
34	1:1.2	0.008	[ChCl][TEA]_2_	5	146	90	3.0	93
35	2:1	0.006	[ChCl][TEA]_2_	5	146	90	3.0	86
36	1:2	0.006	[ChCl][TEA]_2_	5	146	90	3.0	83
37	1:1.2	0.006	[ChCl][TEA]_2_	5	146	70	3.0	63
38	1:1.2	0.006	[ChCl][TEA]_2_	5	146	80	3.0	81
39	1:1.2	0.006	[ChCl][TEA]_2_	5	146	100	3.0	89
40	1:1.2	0.006	[ChCl][TEA]_2_	5	146	110	3.0	87
41	1:1.2	0.006	[ChCl][TEA]_2_	5	146	90	1	78
42	1:1.2	0.006	[ChCl][TEA]_2_	5	146	90	2	86
43	1:1.2	0.006	[ChCl][TEA]_2_	5	146	90	4	90
44	1:1.2	0.006	[ChCl][TEA]_2_	5	146	90	5	90

^a^ HBA: hydrogen bond acceptor, HBD: hydrogen bond donor. The molecular mass (M_DES_) for TEA- based DESs is determined from equation $M_{DES}=x_{HBA}*M_{HBA}+x_{HBD}*M_{HBD}/x_{HBA}+x_{HBD}$ , where M_DES_ is the molecular mass of DES in g.mol^-1^, and are the mole ratio of the HBA and HBD respectively; M_HBA_ and M_HBD_ are the molecular mass of the HBA and HBD in g.mol^-1^, in order.

^b^ Isolated yield.

After determining the optimal reaction conditions, we extensively looked into the efficiency and limitations of this method, focusing on the usage of various phenols. Under optimal conditions, phenol derivatives with electron-donating and electron-withdrawing substituents reacted effectively. It is depicted that electron-rich phenols exhibited higher yields than electron-poor ones (table 3). In addition, iodobenzene, bromobenzene and 1-chloro-4-nitrobenzene were employed as raw materials instead of the other aryl halides such as 1-iodo-4-nitrobenzene and 1-bromo-4-cyanobenzene but the starting materials remained intact after 24 h. It was also decided to use iodo toluenes in the presence of phenol derivatives. The reactions of 2-iodotoluene and 4-iodotoluene with *m*-cresol were carried out under the optimized conditions, and after 5 h it was recognized that these reactions resulted in less than 5% of the desired products (table 3, entries 17 and 18). Then, for exploration of the reactivity of alcohols in this C–O coupling reaction, the reaction of benzyl alcohol with 1-iodo-4-nitrobenzene was run but no product was detected in the reaction mixture after 24 h.

**Table 3.** Pd- catalysed *O*-arylation of phenol derivatives in [ChCl][TEA]_2_.^a^ 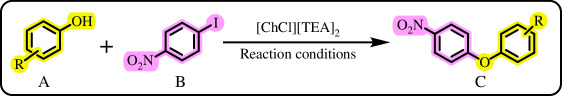

substrate	substrate	product	time (h)	yield ^b^(%)	m.p. ^°^C (lit)
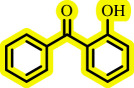	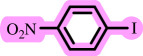	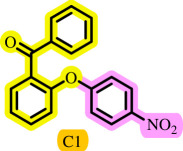	4	79	96–98
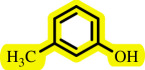	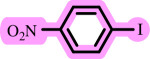	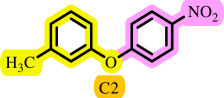	3.5	93	61–63 (61 and 62) [31]
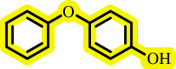	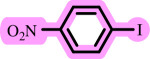	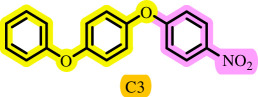		86	89–91
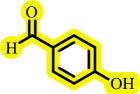	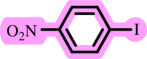	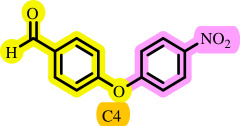	3.5	81	121–123 (122 and 123) [32]
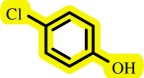	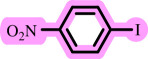	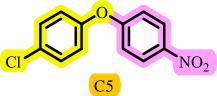	3.5	83	76–78 (76–78) 17]
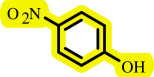	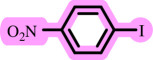	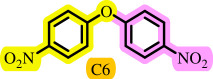	4	79	139–140 (140–142) [33]
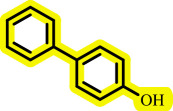	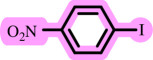	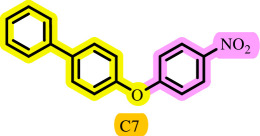	4	84	120 and 121 [33]
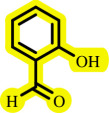	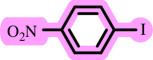	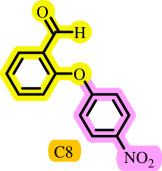	4.15	82	110–112
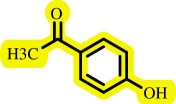	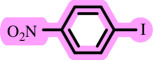	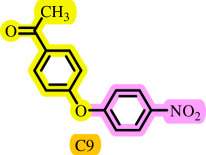	4.45	82	122
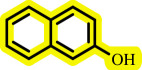	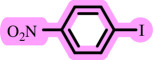	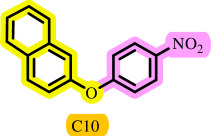	3.5	91	97–98 (96–98) [34]
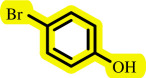	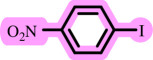	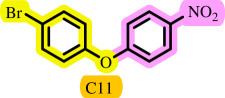	4	84	64–66 [35]
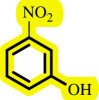	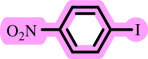	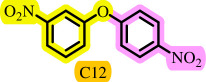	3.5	85	121 and 122 [35]
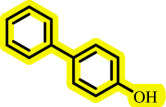	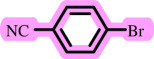	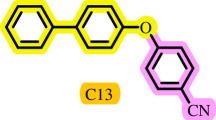	4	79	99–101 (99–102) [36]
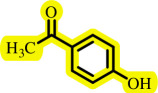	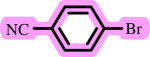	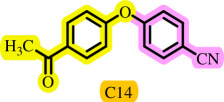	4	76	101–102 (81–84) [37]
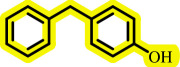	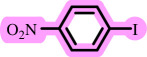	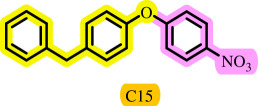	4	88	77–79
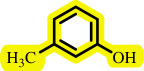	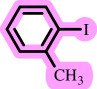	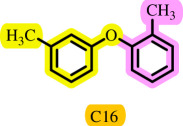	5	<5	— ^c^[38]
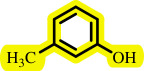	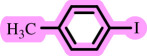	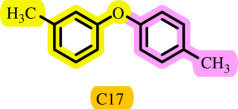	5	<5	— ^c^[39]

^a^ Reaction conditions: phenol derivatives (1.0 mmol), aryl halide (1.2 mmol) and 5 mmol (3.0 ml) of [ChCl][TEA]_2_ (5 mmol, 3.0 ml), Pd/BaSO_4_ (10%, 6 mg).

^b^ Isolated yield.

^c^ Product is oil.

**Scheme 3.**
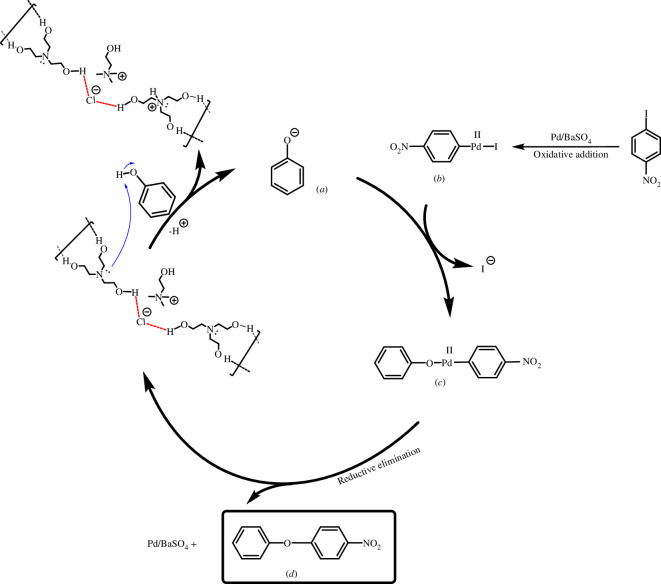
Suggested mechanism for Pd-catalysed *O*-arylation of phenols in [ChCl][TEA]_2_.

After successful *O*-arylation of phenols using [ChCl][TEA]_2_/palladium on barium sulfate as the solvent/catalyst system, based on the chemistry literature and the data achieved in [38], a plausible mechanism is depicted in scheme 3 .

According to the mechanism, the DES activates phenol by the hydrogen abstraction and production of the phenoxide (*a*). Then, displacement of the halide by the phenoxide from the Pd(II) organometal (*b*), which is produced through the oxidative insertion of Pd(0) in the carbon–halogen bond, provides the Pd(II)-organometal (*c*). Finally, the reductive elimination is led to the diaryl ether (*d*) and regeneration Pd/BaSO_4_ and DES (scheme 3).

This proposed mechanism provides a preliminary understanding of the catalytic pathway involved in the *O*-arylation reaction; however, further studies are necessary to investigate the intermediates and transition states involved in this process.

#### 3.1.9. Recovery of deep eutectic solvent ([ChCl][TEA]_2_) as a solvent/catalyst system

**Figure 6.**
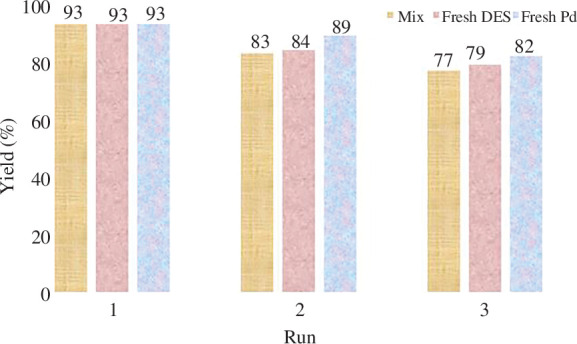
Recycling and re-using of [ChCl][TEA]_2_ and Pd/BaSO4 in the *O*-arylation of *m*-cresol.

The growing interest in recyclable catalysts within the scientific community stems from their extensive industrial usage, cost effectiveness and reduced environmental impact. To address these concerns, we investigated the possibility of recycling the [ChCl][TEA]_2_ solvent/catalyst system in the model reaction. The aim was to develop a sustainable and environmentally friendly approach. The recovery process involved biphasic extraction with ethyl acetate after the completion of the reaction. Subsequently, the DES was recovered through evaporation at 70°C under vacuum conditions for 40 min, allowing its reuse in subsequent reactions. It is worth noting that the recovered [ChCl][TEA]_2_ exhibited a slight decrease in catalytic activity after three consecutive cycles in the model reaction (figure 6). Also, palladium on barium sulfate was simply recovered with filtration, washed twice with ethanol, dried for 2 h at 55°C and then used three times without significant loss of activity. Various tests were done to determine the effectiveness of DES and palladium independently. In this regard, the model’s reaction was carried out once in the presence of recovered DES and fresh palladium, and once in the presence of recovered palladium and fresh DES. The results indicated that palladium has a more significant impact on the reaction process than DES (figure 6). It should be mentioned that the model’s reaction was also examined in the presence of palladium and DES, both of which were recovered. As shown in figure 6, the reaction’s efficiency has dropped with time, but the results are still acceptable.

The ability to recover and re-use [ChCl][TEA]_2_ as a solvent/catalyst system demonstrates its potential for practical applications and highlights its economic and environmental advantages. The slight decline in catalytic activity after repeated use suggests the necessity for further optimization to enhance the recyclability of the DES. Continuous research efforts are required to explore alternative recovery techniques and improve the longevity of the catalyst, thus maximizing its efficiency and minimizing waste generation.

## 4. Conclusion

In this study, we successfully synthesized and characterized a DES, namely [ChCl][TEA]_2_. Various identification techniques, including FT-IR, TGA, DTA, DSC, pH, viscosity, refractive index and conductivity analyses, were employed to confirm the properties and composition of the DES. Subsequently, the application of this DES was considered in the presence of Pd/BaSO_4_ (10%) as a solvent/catalyst system for the *O*-arylation of phenols, which demonstrated remarkable catalytic activity. Notably, the DES ([ChCl][TEA]_2_) exhibited the ability to be collected and re-used for up to three consecutive cycles without significant loss of activity.

To the best of our knowledge, this study represents the first successful report of ligand-free *O*-arylation of phenols without the need for bases in the reaction medium, using a DES. The application of DES in this reaction offers several advantages, including its environmentally benign nature, recyclability and cost effectiveness. The results obtained highlight the potential of DESs as versatile and efficient alternatives to conventional solvents in various catalytic transformations.

## Data Availability

Derived data supporting the findings of this study are available within the article and its supplementary materials [40].
